# Identification and Analysis of *KAS II*, *FAT*, *SAD*, and *FAD* Gene Families in *Hippophae rhamnoides*

**DOI:** 10.3390/plants13243486

**Published:** 2024-12-13

**Authors:** Alexander A. Arkhipov, Ekaterina M. Dvorianinova, Anastasia A. Turba, Roman O. Novakovskiy, Yury A. Zubarev, Pavel A. Predushchenko, Elizaveta A. Sigova, Daiana A. Zhernova, Elena V. Borkhert, Elena N. Pushkova, Chengjiang Ruan, Nataliya V. Melnikova, Alexey A. Dmitriev

**Affiliations:** 1 Engelhardt Institute of Molecular Biology, Russian Academy of Sciences, 119991 Moscow, Russia; arkhipov.aleksandr2.0@gmail.com (A.A.A.); dvorianinova.em@phystech.edu (E.M.D.); anastas.turba@gmail.com (A.A.T.); 0legovich46@mail.ru (R.O.N.); soul_of_the_demon@mail.ru (P.A.P.); sigova.ea@phystech.edu (E.A.S.); zhernova.d@ya.ru (D.A.Z.); sashai@inbox.ru (E.V.B.); pushkova18@gmail.com (E.N.P.); mnv-4529264@yandex.ru (N.V.M.); 2 Federal Altai Scientific Center of Agrobiotechnologies, 656910 Barnaul, Russia; niilisavenko@yandex.ru; 3 Lomonosov Institute of Fine Chemical Technologies, MIREA—Russian Technological University, 119571 Moscow, Russia; 4 Moscow Institute of Physics and Technology, 141701 Moscow, Russia; 5 Faculty of Biology, Lomonosov Moscow State University, 119234 Moscow, Russia; 6 Key Laboratory of Biotechnology and Bioresources Utilization, Ministry of Education, Institute of Plant Resources, Dalian Minzu University, 116600 Dalian, China; ruan@dlnu.edu.cn

**Keywords:** *Hippophae rhamnoides* L., sea buckthorn, fruit development, fatty acids, *KAS II*, *FAT*, *SAD*, *FAD*, gene expression, transcriptomes

## Abstract

*KAS II* (β-ketoacyl-acyl carrier protein (ACP) synthases II), *FAT* (fatty acid thioesterases), *SAD* (stearoyl-ACP desaturase), and *FAD* (fatty acid desaturases) are the vital gene families involved in fatty acid (FA) synthesis in *Hippophae rhamnoides* L. However, information on the number and location of these genes and which ones are key to the formation of FAs in fruit seeds and pulp was not complete. Our study aimed to solve this issue using the available genomic sequences and transcriptome data that we obtained. We compared the protein sequences of sea buckthorn with those of *Arabidopsis thaliana* and checked for the presence of conserved domains. As a result of structure and phylogenetic analyses, 4 *KAS II*, 8 *FAT*, 9 *SAD*, and 12 *FAD* genes were identified in the *H. rhamnoides* genome, which were classified into subfamilies: *KAS II, FATA*, *FATB*, *FAD2*, *FAD3*, *FAD6*, and *FAD7/8*. To analyze the expression of the identified genes, we sequenced the transcriptomes of sea buckthorn seeds and fruit pulp at four development stages, as well as leaves. The analysis revealed representatives of the *FAT*, *SAD*, and *FAD* families with high tissue-and stage-specific expression in seeds and pulp. These genes are likely to play a key role in the biosynthesis of sea buckthorn FAs. The obtained results may help to establish the precise biosynthesis mechanisms of FAs and will promote the breeding of new sea buckthorn varieties that have oil with a defined FA composition.

## 1. Introduction

Sea buckthorn (*Hippophae rhamnoides* L.) is a diploid (2n = 2x = 24) dioecious cultivated berry crop. *H. rhamnoides* genome assemblies of high quality were obtained [1,2,3] and opened up new opportunities for genetic studies of this valuable tree. Sea buckthorn fruits have a unique fatty acid (FA) composition. In sea buckthorn fruit pulp, palmitic (16:0) and palmitoleic (16:1) acids dominate in the FA composition. Meanwhile, oleic (18:1), linoleic (18:2), and linolenic (18:3) acids prevail in sea buckthorn fruit seeds [4,5]. Unsaturated acids are essential in the normal functioning of the human organism [6,7]. The increased ratio of unsaturated FAs in sea buckthorn fruits is beneficial for the human diet [8]. Palmitoleic acid attracts strong interest in industry and medicine [9,10]. The metabolite is less susceptible to oxidation than polyunsaturated FAs and is still essential in human health [11,12]. However, palmitoleic acid is uncommon for plant species, which makes *H. rhamnoides* a unique crop with a high content of this acid [13].

Schemes of FA biosynthesis in sea buckthorn fruits have been presented in several studies [4,14,15] and are, in general, similar to that of *Arabidopsis thaliana* [16]. The pathway starts in plastids with Acetyl-CoA formation. Then, the compound is converted to Malonyl-CoA with acetyl-CoA carboxylase [17,18]. To form a building block for FA biosynthesis, Acetyl-CoA is condensed with Malonyl-CoA with 3-ketoacyl-ACP synthase III (KAS III) and reduced to butyryl-ACP [19]. To form palmitic residue, butyryl-ACP is condensed to 3-oxo-palmitoyl with KAS I, which is reduced to palmitoloyl-ACP [20].

KAS II (beta-ketoacyl-ACP synthase II) is involved in the further chain extension of the 16:0-acyl chain bound to a palmitoloyl-ACP to 18 carbohydrate atoms [21]. The synthesis of the growing FA chain is terminated by the hydrolysis of complex thioesters. This function is performed by the two families of plant thioesterases—FATA (oleoyl-acyl carrier protein thioesterase 1) and FATB (palmitoyl-acyl carrier protein thioesterase). Both enzymes function in chloroplasts. FATA has an affinity to 18:1-ACP; FATB has an affinity to 16- and 18-ACP [22,23].

*SAD* (stearoyl-ACP desaturase) is involved in transforming stearic acid into oleic acid by introducing a double bond in Δ9 position [24]. *FAD2* (delta-12 fatty acid desaturase (fatty acid desaturase 2)) catalyzes the transformation of oleic acid (18:1) into linoleic acid (18:2) by the synthesis of a double bond in Δ12 in the endoplasmic reticulum. Meanwhile, *FAD6* (omega-6 fatty acid desaturase) performs the same function but in chloroplasts [25,26,27]. *FAD3* enzymes convert linoleic (18:2) acid to linolenic acid (18:3) by desaturation in Δ15 position. *FAD3* (acyl-lipid omega-3 desaturase (cytochrome b5)) works in the endoplasmic reticulum, while *FAD7* performs the same conversion (sn-2 acyl-lipid omega-3 desaturase (ferredoxin)) in chloroplasts [25,28,29].

Previous studies identified key gene families determining the main FA composition of sea buckthorn fruits [3,4]. In addition, the differential expression of FA gene sets was analyzed [3,15,30]. Ding et al. analyzed FA gene expression and revealed coordination between several of them and the content of palmitoleic acid. This suggested the genetic basis for the certain content of palmitoleic acid [15]. Yu et al. also studied the expression of sea buckthorn FA genes. Several genes were suggested to play a key role in the synthesis of oleic, linoleic, and linolenic acids [3]. However, there was no targeted search and analysis of the major FA biosynthesis genes in *H. rhamnoides*. In addition, the precise number of active sea buckthorn FA genes remained unclarified. The complete set of genes predetermining FA composition in sea buckthorn fruits remained unknown. However, a detailed analysis of the molecular basis of FA biosynthesis will help us to understand the reasons behind the formation of certain FA ratios in sea buckthorn fruit pulp and seeds. Understanding the mechanisms of lipid biosynthesis can simplify further breeding of sea buckthorn varieties. This can open up novel opportunities for creating quality products from sea buckthorn fruits.

Thus, our study aimed to perform a complex analysis of genes involved in the biosynthesis of sea buckthorn FAs, including their identification, the study of their location on chromosomes and their evolutionary relationships, and an evaluation of their expression to identify the key genes responsible for the synthesis of palmitic, palmitoleic, stearic, oleic, linoleic, and linolenic acids.

## 2. Results

### 2.1. Identification of KAS II, FAT, SAD, and FAD Genes in the Sea Buckthorn Genome

**Figure 1:**
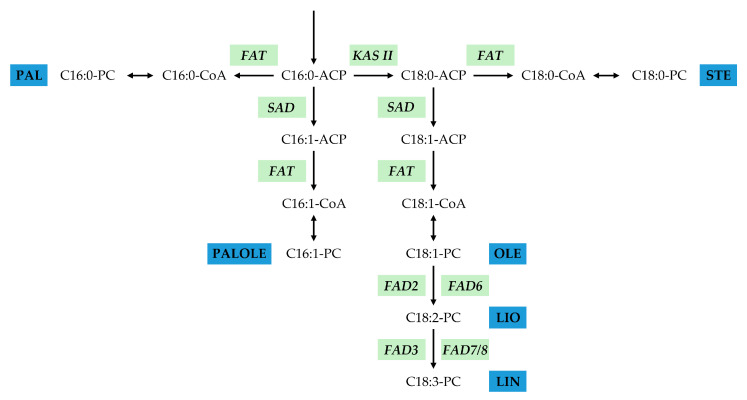
The role of *KAS II*, *FAT*, *SAD*, and *FAD* genes in fatty acid synthesis in *Hippophae rhamnoides*. CoA—coenzyme A, ACP—acyl carrier protein, PC—phosphatidylcholine, PAL—palmitic acid, STE—stearic acid, PALOLE—palmitoleic acid, OLE—oleic acid, LIO—linoleic acid, LIN—linolenic acid.

For our study, we chose genes that are involved in the final steps of FA biosynthesis in sea buckthorn fruits, namely, *KAS II*, *FAT*, *SAD*, and *FAD* (Figure 1). The FA composition of pulp is very different from that of seeds; therefore, the characterization of genes responsible for the synthesis of major FAs of pulp and seeds was of special interest for us. To identify genes of each family, the annotated *H. rhamnoides* genome ID CNP0001846 (https://db.cngb.org/cnsa, accessed on 1 August 2024) was used. We performed the BLASTp analysis of *A. thaliana* proteins of KAS II, FAT, SAD, and FAD families and the search for conserved protein domains of the analyzed gene families. The results of both analyses were matched. Next, we filtered out protein sequences of *H. rhamnoides* according to three criteria: length < 200 a.a., blast identity < 30%, and lacking conserved domains from the corresponding families. The resulting protein candidates were aligned against the *A. thaliana* protein database to check for potential contamination with sequences from closely related gene families. The final set consisted of 4 *KAS II*, 8 *FAT*, 9 *SAD*, and 12 *FAD* genes.

### 2.2. Phylogenetic Analysis and Gene Structure of KAS II, FAT, SAD, and FAD

**Figure 2:**
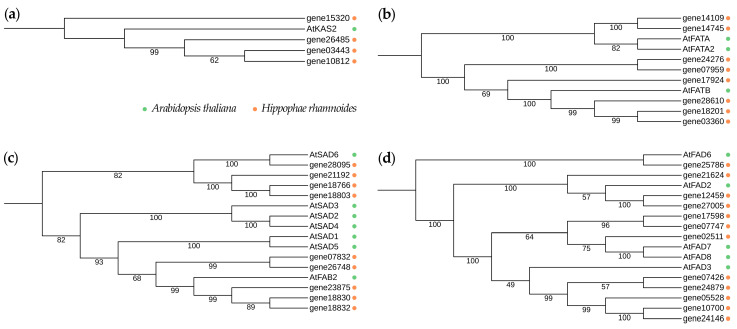
Phylogenetic trees for proteins from the (**a**) KAS II, (**b**) FAT, (**c**) SAD, and (**d**) FAD families of *H. rhamnoides* (orange) and *A. thaliana* (green).

To analyze gene distribution across the studied families and confirm their homology with *A. thaliana* sequences, we constructed phylogenetic trees for each gene family. To analyze the relations of proteins among the KAS II, FAT, SAD, and FAD families, we studied the phylogeny of *A. thaliana* and *H. rhamnoides* sequences (Figure 2).

Two of eight FAT proteins (14745 and 14109) of sea buckthorn were clustered with FATA of *A. thaliana*, while proteins 24276, 07959, 17924, 28610, 18201, and 03360 were clustered with FATB of *A. thaliana* (Figure 2b). Nine SAD proteins of *H. rhamnoides* were divided into three groups: (1) 18830, 18832, and 23875; (2) 07832 and 26748; and (3) 28095, 21192, 18766, and 18803 (Figure 2c). In the FAD family, three proteins of *H. rhamnoides* (21624, 12459, 27005) were grouped with FAD2 of *A. thaliana*. Protein 25786 of sea buckthorn grouped with FAD6 of *A. thaliana*. Five proteins of sea buckthorn (07426, 24879, 05528, 10700, and 24146) were grouped in one cluster with *A. thaliana* FAD3. *H. rhamnoides* proteins 02511, 17598, and 07747 were clustered with FAD7 and FAD8 of *A. thaliana* (Figure 2d). Thus, the identified sea buckthorn proteins were classified into subfamilies: 4 KAS II, 2 FATA, 6 FATB, 9 SAD, 3 FAD2, 1 FAD6, 5 FAD3, and 3 FAD7/8.

### 2.3. Characterization of KAS II, FAT, SAD, and FAD Genes in Sea Buckthorn Genome

We identified four *KAS II*, two *FATA*, six *FATB*, nine *SAD*, three *FAD2*, one *FAD6*, five *FAD3*, and three *FAD7/8* genes in the *H. rhamnoides* genome (Table 1, Supplementary File S1: Table S1). The genes were named according to their homology to *A. thaliana* genes and according to transcript IDs from the annotation of the *H. rhamnoides* genome, e.g., *Gene* (transcript ID).

Exon–intron, conserved domain, and motif structures of the studied sea buckthorn genes and their distribution on chromosomes are present in Supplementary File S2: Figures S1–S4. Motif sequences are given in Supplementary File S1: Table S2. Five of the nine identified *SAD* genes had three exons. *SAD* (18803) and *SAD* (28095) had two exons, while *SAD* (18766) and *SAD* (18832) had eight and five exons, respectively. The majority of the *SAD* genes had a similar motif structure. Eight of the nine *SAD* proteins had a structure with motifs 5, 3, 10, 1, 7, 2, and 8 in tandem. In *SAD* (18832), only motifs 5 and 2 were present. *SAD* (18766) had an unexpectedly long sequence and a strikingly different exon–intron structure compared to the other analyzed *SAD* genes. BLASTn analysis of *SAD* (18766) against the NCBI core nucleotide database demonstrated its alignment with the IQ-DOMAIN 14 sequence but not plant *SAD* sequences. However, blasting the end of the *SAD* (18766) sequence in the same database showed its alignment with many plant *SAD* sequences. This suggested an error (two different genes under one ID were combined) in the annotation of *SAD* (18766) in the *H. rhamnoides* genome ID CNP0001846.

*KAS II* genes had 13 exons. All *KAS II* representatives were characteristic of motifs 2, 8, 1, 3, 6, and 4. *KAS II* (15320) did not include motifs 9, 5, and 7, which were present in other *KAS II* genes.

All *FAT* genes had 6–7 exons, except for *FATB* (28610), which had 11 exons. All *FAT* genes were characteristic of motifs 1, 3, 10, 2, 7, and 6. All *FATA* genes also included motif 8, while *FATB* also had motifs 9 and 5. *FATB* (17924) lacked both of these motifs. *FATB* (28610) had the longest sequence; however, the BLASTn analysis showed that errors in the annotation of the *H. rhamnoides* genome ID CNP0001846 were probable, only the second half of the gene corresponded to the complete plant *FAT* sequences.

The genes of the *FAD2* subfamily had two exons, except for *FAD2* (27005) lacking introns. All *FAD2* genes had motifs 4, 6, 3, 8, 9, 1, and 10 in tandem. For *FAD2* (12459) and *FAD2* (21624), there were probably errors in the annotation of *H. rhamnoides* genome assembly ID CNP0001846, particularly the presence of introns. *FAD3* genes had a similar structure, comprising eight exons. *FAD3* (24146) had an overlong sequence for the first intron, and its BLASTn analysis suggested a potential error in the annotation of this gene in the *H. rhamnoides* genome ID CNP0001846. *FAD7/8* (further referred to as *FAD7*) had 7–8 exons, while *FAD6* had the greatest number of exons (10) among all the identified *FAD* genes. *FAD6* had only two motifs—3 and 6. *FAD3* and *FAD7* were characteristic of motifs 4, 6, 3, 7, 2, 1, and 9.

Then, we studied the properties of the sequences from the analyzed gene families and proteins encoded by them. The information on the studied genes (gene family, gene ID, gene location, number of exons, length of amino acid sequence, protein molecular mass (kDa), protein isoelectric point (pI), protein instability index (II), and gene CDS) is specified in Table 1 and Supplementary File S1: Table S1.

Our analysis demonstrated that the identified genes were unevenly distributed across eleven of the twelve chromosomes of the sea buckthorn genome (Supplementary File S2: Figure S1). Three chromosomes encoded the biggest numbers of genes: chromosome 1 (five genes), chromosome 5 (six genes), and chromosome 11 (nine genes).

We observed duplication between *FAD3* and *FAD7* genes. In addition, the duplicates from the *FATB* and *KAS II* subfamilies grouped together. The same phenomenon was observed for *SAD* and *FAD3*. They grouped in pairs in four of five cases: a pair of *SAD* and *FAD3* copies was located on each of chromosomes 5 and 8, and two pairs of *FAD3* and *SAD* copies were located on chromosome 11. Only *FAD3* (24879) was an exception. This gene was located on chromosome 2, which had no *SAD* genes.

**Figure 3:**
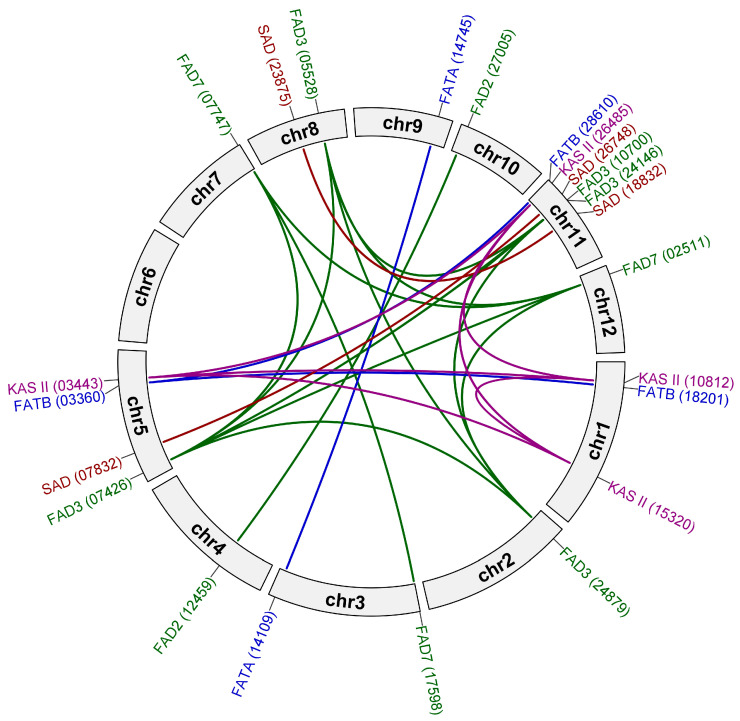
Location of duplicated *KAS II*, *FAT*, *SAD*, and *FAD* genes on twelve chromosomes of *H. rhamnoides*.

Since the studied genes are prone to having multiple copies, gene duplication was confirmed with MCScanX and visualized with TBtools. We identified 1 tandem (adjacent) and 25 segmental (matched in syntenic blocks) duplications (Figure 3). Among the genes of *SAD* families, we found two segmental duplications: (*SAD* (18832); *SAD* (23875)) and (*SAD* (26748); *SAD* (07832)).

In the *FAD2* subfamily, one pair of *FAD2* genes was duplicated: (*FAD2* (27005); *FAD2* (12459)). In the *FAD3* subfamily, all five genes proved to be duplicates. Among them, one gene pair (*FAD3* (10700); *FAD3* (24174)) existed as a tandem duplication and was located on chromosome 11. In addition, we observed that a range of *FAD3* genes had duplicates in the *FAD7* subfamily. Also, in the *FAT* family, two genes were a pair of duplicates: (*FATA* (14109); *FATA* (14745)). Also, three genes from the *FATB* subfamily were duplicated on different chromosomes. All *KAS II* genes were duplicated. Using the in-built function of Ka/Ks Calculator in TBtools, we calculated nonsynonymous substitution rates Ka, synonymous substitution rates Ks, and the ratio of nonsynonymous substitution rates to synonymous substitution rates (Ka/Ks) for duplicated genes. For the tandem duplication, Ka/Ks was 0.99, and for the segmental ones, the value was in the range of 0.11–0.35 (Supplementary File S1: Table S3).

The properties of proteins encoded by the analyzed gene families can reveal the similarity with *A. thaliana* enzymes and suggest their functionality. The length of protein sequences encoded by *SAD* genes (*SAD* (18766) was not taken into account because of the critical error in the annotation) varied from 281 a.a. to 397 a.a., corresponding to SAD (18832) and SAD (28095), respectively. The molecular masses of SAD proteins varied from 32.1 kDa to 45.4 kDa, with SAD (18832) at the lower boundary and SAD (26748) at the higher boundary. The theoretical isoelectric point (pI) in proteins varied from 5.9 (for three SAD proteins) to 8.6 (for SAD (21192)). Thus, six and two SAD representatives were acid (pI < 7) and alkyl (pI > 7), respectively. Determining the instability index demonstrated that the majority of SAD proteins were stable (five from eight, II < 40).

FAT proteins were separated into two subfamilies—FATA and FATB. The length of FAT proteins was from 378 a.a. for FATB (17924) to 443 a.a. for FATB (03360) (FATB (28610) was not taken into account because of the critical error in the annotation). The molecular masses were in the range of 42.8 kDa (for FATA (14745)) and 57.3 kDa (for FATB (07959)). The theoretical isoelectric point in FAT proteins varied from 6.6 (for two FATB proteins) to 9.4 (for FATB (24276)). Two and five FAT representatives were acid (pI < 7) and alkyl (pI > 7), respectively. Three of the seven FAT proteins were predicted stable: FATB (17924), FATA (14745), and FATB (07959) were stable.

The length of KAS II proteins varied from 563 a.a. (for KAS II (03443) and KAS II (26485)) to 571 a.a. (for KAS II (10812)). The molecular masses of KAS II proteins varied from 60.3 kDa (for KAS II (26485)) to 61.8 kDa (for KAS II (15320)). The theoretical isoelectric point varied from 6.9 to 8.8, corresponding to KAS II (03443) and KAS II (15320), respectively. One and three KAS II representatives were acid (pI < 7) and alkyl (pI > 7), respectively. All KAS II proteins were unstable.

### 2.4. Expression Profiles at the Four Stages of Sea Buckthorn Fruit Development

**Figure 4:**
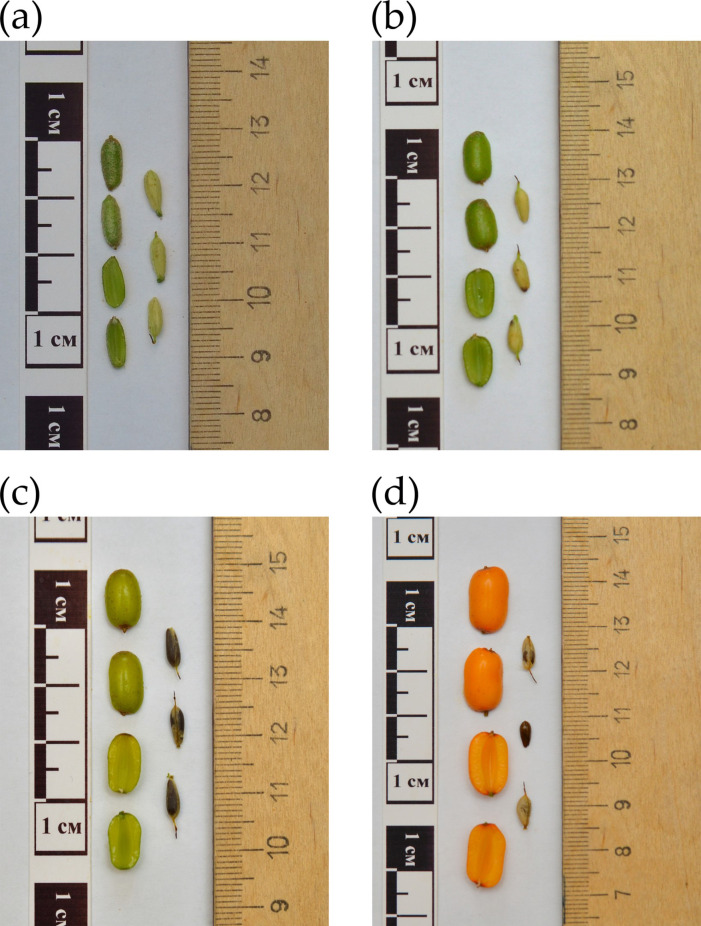
*H. rhamnoides* fruits and seeds at different time points of gathering: (**a**) first stage—30 June 2024 (hypanthium to seed disruption), (**b**) second stage—14 July 2024 (the supposed start of fat accumulation), (**c**) third stage—2 August 2024 (active fat accumulation in hypanthium), (**d**) fourth stage—29 August 2024 (the period of full maturation).

To analyze the level of expression of the studied genes, we sequenced the transcriptomes of seeds and pulp of the variety Yantarnaya yagoda gathered at four stages of fruit development: ~7 weeks after pollination (WAP) (hypanthium to seed disruption), ~9 WAP (the supposed start of fat accumulation), ~12 WAP (active fat accumulation in hypanthium), and ~16 WAP (the period of full maturation) (Figure 4). At ~7 WAP, leaves were also gathered.

**Figure 5:**
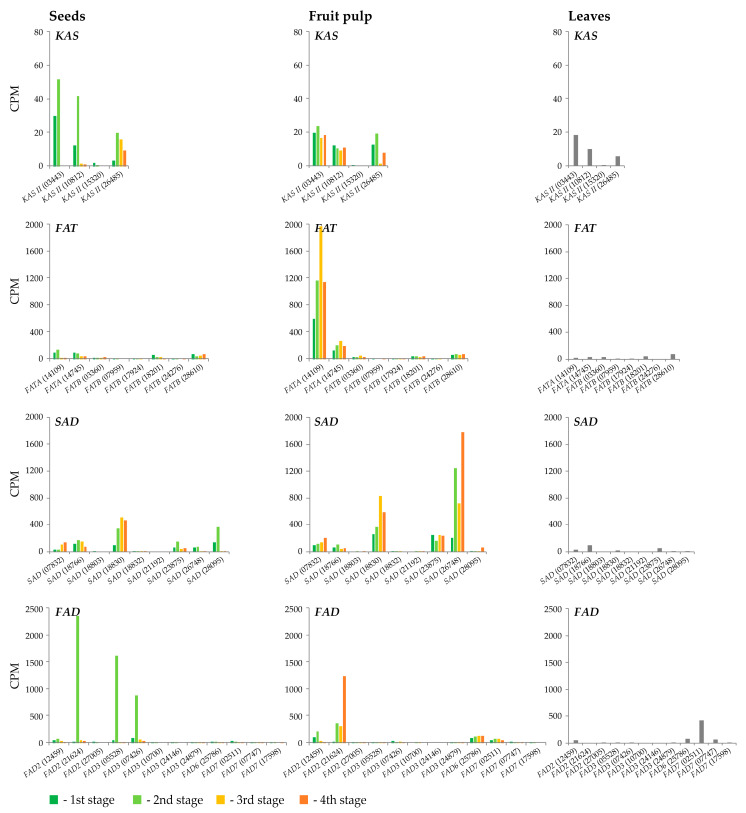
Expression profiles of *KAS II*, *FAT*, *SAD*, and *FAD* genes in fruit seeds and pulp at four development stages and leaves of *H. rhamnoides*.

*KAS II* genes had low expression levels (Figure 5, Supplementary File S1: Table S4). The most highly expressed were *KAS II* (03443) and *KAS II* (10812)—maximum expression levels were revealed in seeds at the second stage. In addition, the dynamics in expression levels during fruit development was observed for these genes. Expression levels of all *KAS II* genes in fruits were not significantly higher than in leaves.

In the *FAT* family, the expression of all *FATA* genes in the pulp was much higher than in seeds (Figure 5, Supplementary File S1: Table S4). *FATA* (14109) and *FATA* (14745) were expressed at all stages of pulp ripening and reached their maximum level at the third stage. However, at the fourth stage of development, the expression decreased. *FATA* (14109) had a six times greater expression than *FATA* (14745) on average. In seeds, *FATA* (14109) and *FATA* (14745) had a higher expression at the first and second stages of development than at the third and fourth stages. In leaves, the expression of these genes was relatively low. Thus, *FATA* (14109) and *FATA* (14745) should have the greatest impact on the synthesis of FAs in sea buckthorn pulp. *FATB* genes had low expression in pulp, seeds, and leaves. For *FATB* (17924), *FATB* (24276), and *FATB* (07959), it was close to zero. However, *FATB* (18201) expression gradually decreased in seeds with ripening although it was not high.

In the *SAD* family, six out of nine *SAD* genes were expressed at considerable levels at four stages of fruit development (Figure 5, Supplementary File S1: Table S4). *SAD* (18803), *SAD* (18832), and *SAD* (21192) genes had low expression levels at all stages in seeds, pulp, and leaves. *SAD* (26748) had the highest expression in pulp among the *SAD* genes, while its expression in seeds was much lower. Thus, the expression of this gene was tissue-specific. At the fourth stage of development, its expression in pulp was ~100 times higher than in seeds. In contrast, *SAD* (28095) had a higher expression in seeds than in pulp at the first and second stages of ripening. However, at the third stage, its expression in seeds decreased drastically (~200 times). The expression dynamics of other *SAD* genes (*SAD* (07832), *SAD* (23875), *SAD* (18766), *SAD* (18830)) was similar for sea buckthorn seed and pulp. For *SAD* (18830), which was the most highly expressed among the *SAD* genes in seeds, the expression level increased from the first to third stages and then slightly decreased in both seeds and pulp. At the same time, the expression level of *SAD* (18830) was low in leaves. Therefore, the *SAD* (18830) gene is likely important for oleic acid synthesis in sea buckthorn fruits, especially in seeds. Thus, *SAD* (26748), *SAD* (18830), and *SAD* (28095) may have the most impact on the synthesis of oleic acid in sea buckthorn fruits.

In the *FAD2* family, *FAD2* (21624) had the highest expression among all the *FAD* genes in pulp and seeds, while it was not expressed in leaves (Figure 5, Supplementary File S1: Table S4). Its mRNA level was low in pulp at the first development stage but increased further and reached its maximum at the fourth stage. In seeds, *FAD2* (21624) had the highest expression level at the second stage of development (~50 times higher than at other three stages). A significant expression level was also revealed for *FAD2* (12459) in pulp (first and second stages), while at the third and fourth stages, its level was reduced. Thus, *FAD2* (21624) is likely to primarily contribute to the synthesis of linoleic acid in both seeds and pulp, while *FAD2* (12459) is also implicated in this process in pulp.

*FAD3* (07426) and *FAD3* (05528) demonstrated tissue-specific patterns, as their expression was significantly higher in seeds than in pulp. Thus, *FAD3* (07426) and *FAD3* (05528) are likely the key genes in linolenic acid synthesis in sea buckthorn seeds.

*FAD6* (25786) and *FAD7* (02511) were several times more highly expressed in pulp than in seeds. However, their expression levels were also significant in leaves (for *FAD7* (02511), it was ~10 times higher than in fruits). The *FAD7* (07747) expression level was also higher in leaves than in fruits. It is likely that the *FAD6* and *FAD7* genes are not major contributors in the synthesis of linoleic and linolenic acids in sea buckthorn fruits.

## 3. Discussion

FAs in plant oil are beneficial products in many industries. However, the properties of the raw material have a direct impact on the effectiveness of manufacturing the product. An increased content of unsaturated FAs in the target plant part can be achieved with molecular-based methods of selection. Thus, dissecting the pathways of FA biosynthesis is mandatory in the successful targeting of genomic features encoding these valuable metabolites. Sea buckthorn fruits are rich in bioactive compounds, e.g., vitamins, carotenoids, and FAs [31]. Linoleic and α-linolenic acids are beneficial to human health [4]. Thus, the ratio of ω-3 to ω-6 FAs determines the value of sea buckthorn oil for many applications, e.g., in the food and cosmetic industries and pharmaceutics [32]. Thus, to reach higher concentrations of unsaturated FAs, genes involved in their synthesis can be targeted. The key families of the FA synthesis genes had been discovered [4]. However, there was a gap in knowledge regarding the genes with the greatest impact on the accumulation of certain sea buckthorn FAs.

In our work, we identified and characterized the key genes involved in the final steps of synthesis of the main FAs in sea buckthorn fruits: palmitic, palmitoleic, stearic, oleic, linoleic, and linolenic acids. Using the available genomic data, we searched for the sequences of the *KAS II*, *FAT*, *SAD*, and *FAD* families and studied their role in FA biosynthesis using our transcriptome data. Thus, 4 *KAS II*, 8 *FAT*, 9 *SAD*, and 12 *FAD* genes were identified. Two previous studies on *H. rhamnoides* allowed the identification of 16 *SAD* genes, 12 *FAD* genes, 6 *FAT* genes, and 25 *KAS* genes [15] or 5 *SAD* genes, 11 *FAD* genes, 10 *FAT* genes, and 9 *KAS* genes [3]. However, the characteristics of the structures of these genes were absent. In addition, these sequences were not compared with the corresponding ones for other plant species. We characterized the properties of these genes and identified their genome location. *SAD* genes catalyze the conversion of stearic acid to oleic acid. The number of *SAD* genes in sea buckthorn is greater than that for certain plants cultivated for oil, e.g., flax (four *SAD* genes) [33,34] or walnut (nine *SAD* genes) [35]. Flax has 21 genes from the *FAD* family, while cultivated olive has 40 *FAD* genes [34,36]. These numbers are greater than that for *H. rhamnoides*. In wild types of a plant, the greater number of FA genes can be due to evolutionary pressure, helping the plant to adapt to a harsh environment. In addition, the difference in the number of genes points to the unique history of duplications, as well as the underlying causes for these events.

An analysis of gene structure revealed that certain genes had extremely long sequences that were significantly different from the other representatives of their families. BLASTn analysis revealed the errors in the annotation of the *H. rhamnoides* genome assembly ID CNP0001846. We provided the proper comments for such genes in the Section 2.3 of Results. When studying these genes, it is important to take possible errors into account.

We identified 26 gene duplications in the studied gene families. Segmental duplications were located on eleven of the twelve chromosomes of *H. rhamnoides*. Such a character suggests the expansion of the gene families involved in FA ratio regulation. One tandem duplication was present in the *FAD* family (*FAD3* (10700) and *FAD3* (24174)) and was located on chromosome 11. In addition, we observed the simultaneous duplications of *FATB* and *KAS II* genes, as well as *FAD3* and *SAD* genes. These events may suggest the genes’ equal significance in the plant’s survival or that they could have been simultaneously selected due to their biological functions.

The analysis of the physical and chemical properties of the studied *SAD* genes of *H. rhamnoides* demonstrated that the length of amino acid sequences, their molecular masses, and the pI values were significantly different. These differences might be due to their functionality and functional diversity. However, the properties of *FAD* and *FAT* genes were very similar within their subfamilies. The analysis of the exon–intron structure also proved this fact. *SAD* genes had significantly differing number of exons. Meanwhile, *FAD* and *FAT* genes had a similar number of exons within the same subfamilies. The same was true for the *KAS II* family. The analysis of the motif structure allowed us to confirm the distribution among the gene families. The majority of the analyzed genes from the same subfamilies had similar motif structures, allowing us to attribute each gene to a certain subfamily.

To evaluate the significance of the role of the studied genes in FA synthesis, we conducted differential gene expression analysis. We obtained transcriptome data for seeds and pulp at four stages of fruit development and for leaves. The expression analysis of FA genes revealed tissue-specific patterns for some of them.

*FATA* (14109) and *FATA* (14745) had high expression only in sea buckthorn pulp, with *FATA* (14109) having the highest expression level among all the analyzed *FAT* genes, indicating its role in FA synthesis in sea buckthorn fruits.

*KAS II* genes had low expression levels in sea buckthorn fruits and leaves, which could indicate their low importance in the synthesis of FAs in sea buckthorn fruits. However, Ding at al. revealed the association of the expression level of *KAS II* with the content of palmitoleic acid [15]. Possibly, differences in the studied genotypes had an impact on the expression of *KAS II* genes.

In the *SAD* family, *SAD* (26748) had tissue-specific expression in pulp. Meanwhile, *SAD* (28095) had tissue-specific expression in seeds and was actively expressed at the first and second stage of fruit ripening. This was probably due to oleic acid accumulation at earlier stages of seed development when fruits had greenish-yellow color [4]. However, oleic acid accumulation in fruit pulp continued during the whole period of development. In pulp, *SAD* (18830) and *SAD* (26748) had the highest levels of expression. Thus, these genes likely made the most significant contribution to the conversion of stearic acid to oleic acid in pulp. In seeds, high expression was characteristic of *SAD* (18830) and *SAD* (28095). Yu et al. studied the expression of five *SAD* genes at the 7th, 9th, and 11th weeks after anthesis. Similarly to our results, two *SAD* genes had the highest expression in seeds (Hr1g3285 and Hr9g0203), and two other *SAD* genes had the highest expression in pulp (Hr9g0203 and Hr11g2328). The researchers observed high expression of one of the three *FAD2* genes (Hr1g1059) in both seeds and pulp [3]. In our work, *FAD2* (21624) had the highest expression level at the second stage of development in seeds and the fourth stage in pulp among the *FAD2* genes. Thus, our results were again similar to those of other researchers. *FAD2* (21624) probably makes the greatest contribution to the synthesis of linolenic acid from oleic acid in sea buckthorn fruits. Among the *FAD3* genes, *FAD3* (07426) and *FAD3* (05528) had high expression levels in seeds (at the second stage of development). However, none of the *FAD3* genes had high expression in pulp. *FAD3* genes are responsible for the conversion of linoleic acid into linolenic acid. Thus, our result is very natural, since sea buckthorn fruits contain a lot of linolenic acid in the seeds and almost none in the pulp. It should be noted that the *FAD6* and *FAD7/8* genes are responsible for the conversion of oleic acid to linoleic acid and linoleic acid to linolenic acid, respectively, but unlike the *FAD2* and *FAD3* genes, they function in plastids. The expression of the *FAD6* and *FAD7/8* genes in sea buckthorn fruits was relatively low. Moreover, these genes had similar or even higher expression in sea buckthorn leaves. It is likely that *FAD6* and *FAD7/8* do not contribute significantly to the synthesis of linoleic and linolenic acids in sea buckthorn fruits but are important in FA synthesis in other organs.

In addition to the gene families that we studied, a number of other genes contribute to FA synthesis in sea buckthorn fruits [4,14,15]. Apart from genes directly involved in FA synthesis pathways, a number of other genes are likely to be associated with FA accumulation in sea buckthorn fruits. For example, the expression of the phosphoenolpyruvate carboxylase gene was associated with the expression of genes from the FA synthesis pathway in rice [37]. For the identification of genes that are also important in FA accumulation in sea buckthorn fruits, it is pertinent to search for genes that have similar expression profiles to those of the key *FAT*, *SAD*, and *FAD* genes, which, according to our transcriptome analysis, may be major contributors to FA synthesis in the seeds and pulp of sea buckthorn fruits.

In this work, we identified key genes involved in the biosynthesis of sea buckthorn FAs. The analysis of their structure and phylogeny allowed us to assign the analyzed sequences to gene subfamilies. The data on their properties and expression will simplify further studies on the evolution, domestication, and adaptation of *H. rhamnoides*. Our information on the key genes in FA biosynthesis can assist in the creation of new sea buckthorn varieties with a known FA composition.

## 4. Materials and Methods

### 4.1. Gene Identification in the H. rhamnoides Genome

The *H. rhamnoides* genome, structural annotation in the GFF format, and the corresponding protein sequences were downloaded from the CNGB Nucleotide Sequence Archive (https://db.cngb.org/cnsa, accessed on 1 August 2024; ID CNP0001846) [1]. To identify the target gene families (*KAS II*, *FAT*, *SAD*, and *FAD*), the relevant protein sequences of *A. thaliana* (Supplementary File S1: Table S5) were downloaded from The Arabidopsis Information Resource—TAIR (https://www.arabidopsis.org, accessed on 1 August 2024) [38]. These sequences were used as queries in BLASTp [39] with an E-value ≤ 10^−10^ and the list of relevant *H. rhamnoides* proteins was obtained. In addition, Hidden Markov Models (HMMs) of the studied gene families were downloaded from the Pfam database (https://www.ebi.ac.uk/interpro/, accessed on 1 August 2024) [40] and used as queries in HMMER 3.0 (http://hmmer.janelia.org/, accessed on 1 August 2024) to search for candidate proteins among all the downloaded *H. rhamnoides* sequences. Protein sequences lacking any of the searched domains of the corresponding gene family were filtered out. Proteins selected by BLAST analysis were matched with those chosen by domain search. *H. rhamnoides* proteins with length <200 a.a. and identity with *A. thaliana* sequences <30% were excluded from the merged list. The presence of conserved domains in the remaining proteins was verified using NCBI CD-Search [41]. The resulting protein candidates were aligned against the *Arabidopsis* protein database using BLASTp to check for potential contamination with sequences from closely related gene families. To analyze the final set of sequences and verify the absence of stop codons within the ORF, the online tool ExPASy (ProtParam and Translate resources) was used [42].

### 4.2. Location on Chromosomes, Gene Duplication, and Covariant Analysis

Gene location was visualized with MapGene2Chrom (http://mg2c.iask.in/mg2c_v2.1/, accessed on 1 August 2024) [43]. Gene coordinates were taken from the annotation file of *H. rhamnoides* (Nucleotide Sequence Archive; https://db.cngb.org/cnsa, accessed on 1 August 2024; ID CNP0001846). MCScanX [44] was used to verify gene duplication (default parameters). Intraspecies gene duplicates in *H. rhamnoides* were analyzed with Advanced Circos (TBtools) [45]. The KaKs_Calculator tool was used to calculate the synonymous substitution rates (Ks), nonsynonymous substitution rates (Ka), and, for the duplicated genes, the ratio of nonsynonymous substitution rates to synonymous substitution rates (Ka/Ks) [46].

### 4.3. Structure of Genes and Conserved Motifs

To predict conserved protein motifs, MEME v5.1.1 [47] was used with the following parameters: the minimal length, maximum length, and maximum number of motifs were set to 6 a.a., 100 a.a., and 10, respectively. The visualization of the exon–intron gene structure and the location of conserved motifs in proteins were performed with the ggplot2 R package (https://ggplot2.tidyverse.org/, accessed on 1 August 2024).

### 4.4. Multiple Alignment and Phylogenetic Analysis

To study evolutionary relationships in gene families, phylogenetic trees were constructed. Full protein sequences of *A. thaliana* and *H. rhamnoides* were used. Multiple alignment of sequences was performed with MAFFT v7.525. For phylogenetic tree construction, the Neighbor Joining (NJ) method was used (1000 bootstrap replicates)—RapidNJ 2.3.2. iTOL v6.9.1 was used for phylogenetic tree visualization.

### 4.5. Expression Analysis

The gene expression of FA genes was studied in sea buckthorn fruit pulp, seeds, and leaves. Materials of the variety Yantarnaya yagoda were gathered at the Federal Altai Scientific Center of Agrobiotechnologies (Barnaul, Russia). Time points for fruit gathering were chosen according to the information on different periods of fat accumulation in sea buckthorn fruits: ~7 weeks after pollination (WAP) (hypanthium to seed disruption), ~9 WAP (the supposed start of fat accumulation), ~12 WAP (active fat accumulation in hypanthium), and ~16 WAP (the period of full maturation). The fruit was separated into two parts—the seed and the soft part of the fruit (pulp) were gathered separately. Leaves were collected at the 7 WAP stage. The samples were frozen in liquid nitrogen and stored at −70 °C until RNA isolation took place.

RNA isolation was performed from the pools of samples: 30 seeds and 24 pulp samples for the first stage of ripening, 24 seeds and 18 pulp samples for the second stage, 24 seeds and 16 pulp samples for the third stage, 20 seeds and 8 pulp samples for the fourth stage, and 4 leaves. The material was homogenized in liquid nitrogen with an electric drill. For RNA isolation, ~100 mg of a sample was taken, and 1.5 mL of cold acetone (90% or 80%) was added. Two successive washes to remove excessive oils were performed with 90% and 80% acetone. CTAB buffer (100 mM Tris-HCl pH 9.5, 2% CTAB, 2 M NaCl, 2% PVP K30, 25 mM EDTA) was used for cell lysis. Then, purification with CleanRNA Standard kit (Evrogen, Moscow, Russia) was performed according to the manufacturer’s protocol. The concentration of the extracted RNA was evaluated with fluorometry (Qubit 4.0; Thermo Fisher Scientific, Waltham, MA, USA), and its quality was assessed with electrophoresis in 2% agarose gel. RNA libraries were prepared with QIAseq Stranded RNA Library Kit (Qiagen, Chatsworth, CA, USA) according to the manufacturer’s protocol. Sequencing was performed on a NextSeq 2000 instrument (Illumina, San Diego, CA, USA) with a read length 50 + 50 bp. Raw reads were processed and mapped to the *H. rhamnoides* genome (CNGB Nucleotide Sequence Archive; https://db.cngb.org/cnsa/, accessed on 1 August 2024; ID CNP0001846) with PPLine [48]. Differential gene expression was analyzed with RTrans (https://github.com/gskrasnov/RTrans, accessed on 1 August 2024).

## 5. Conclusions

In this work, we identified and characterized genes within the *KAS II*, *FATA*, *FATB*, *SAD*, *FAD2*, *FAD3*, *FAD6*, and *FAD7/8* families of *H. rhamnoides*, which are involved in the final stages of FA synthesis. Transcriptome analysis of sea buckthorn seeds, fruit pulp, and leaves revealed genes with high tissue-specific and ripening-stage-specific expression, suggesting that they are key to FA biosynthesis in seeds and pulp, which have drastically different FA compositions. Our study enhances the understanding of the biological mechanisms of FA synthesis and narrows down the search for potential target genes in sea buckthorn breeding. Our data will facilitate the effective creation of improved sea buckthorn varieties that have oil with the desired FA composition.

## Figures and Tables

**Table 1:** Characteristics of the *H. rhamnoides* genes from the *KAS II*, *FAT*, *SAD*, and *FAD* families.

Gene Name	Sequence ID	Chr. Location (Strand)	Start	End	Ex.	Protein Length (a.a.)	MW (kDa)	pI	II
*KAS II* (03443)	Hiprha1gene03443	chr5 (+)	50795429	50800189	13	563	60.6	6.9	41.7
*KAS II* (10812)	Hiprha1gene10812	chr1 (−)	10100481	10106836	13	571	61.6	8.2	46.9
*KAS II* (15320)	Hiprha1gene15320	chr1 (+)	56813452	56818307	13	568	61.8	8.8	43.0
*KAS II* (26485)	Hiprha1gene26485	chr11 (+)	2378857	2384863	13	563	60.3	7.2	46.6
*FATA* (14109)	Hiprha1gene14109	chr3 (+)	70531992	70535297	7	381	43.2	6.6	38.1
*FATA* (14745)	Hiprha1gene14745	chr9 (−)	40902521	40905529	7	380	42.8	6.6	43.0
*FATB* (03360)	Hiprha1gene03360	chr5 (−)	48119192	48127428	7	443	49.3	7.6	32.8
*FATB* (07959)	Hiprha1gene07959	chr8 (−)	41928708	41931484	6	403	57.3	7.7	46.4
*FATB* (17924)	Hiprha1gene17924	chr4 (+)	3525474	3527769	6	378	43.3	8.5	42.9
*FATB* (18201)	Hiprha1gene18201	chr1 (+)	12284001	12286875	6	421	46.8	7.7	36.3
*FATB* (24276)	Hiprha1gene24276	chr5 (+)	60318530	60322010	7	408	46.0	9.4	39.5
*FATB* (28610) *	Hiprha1gene28610	chr11 (−)	844598	856744	11	691	76.3	6.3	50.6
*SAD* (07832)	Hiprha1gene07832	chr5 (−)	14947622	14953246	3	393	44.9	5.9	40.2
*SAD* (18766) *	Hiprha1gene18766	chr11 (+)	21045762	21052997	8	936	105.8	9.8	53.1
*SAD* (18803)	Hiprha1gene18803	chr11 (+)	21116761	21117840	2	308	35.3	5.9	39.7
*SAD* (18830)	Hiprha1gene18830	chr11 (+)	21159091	21162852	3	396	45.2	6.1	41.4
*SAD* (18832)	Hiprha1gene18832	chr11 (+)	21081792	21083867	5	281	32.1	8.5	37.6
*SAD* (21192)	Hiprha1gene21192	chr5 (−)	6969	8437	3	383	43.9	8.6	38.0
*SAD* (23875)	Hiprha1gene23875	chr8 (+)	24166998	24170533	3	396	45.2	6.7	35.8
*SAD* (26748)	Hiprha1gene26748	chr11 (+)	9512943	9517500	3	396	45.4	5.9	42.4
*SAD* (28095)	Hiprha1gene28095	chr1 (−)	1003507	1005156	2	397	44.6	6.8	27.6
*FAD2* (12459) *	Hiprha1gene12459	chr4 (−)	22789978	22791126	2	370	42.8	8.9	43.8
*FAD2* (21624) *	Hiprha1gene21624	chr1 (+)	70347395	70349092	2	390	44.9	8.6	42.4
*FAD2* (27005)	Hiprha1gene27005	chr10 (−)	3227260	3228453	1	397	46.0	8.9	43.9
*FAD3* (05528)	Hiprha1gene05528	chr8 (−)	36136884	36139154	8	409	47.8	8.9	32.4
*FAD3* (07426)	Hiprha1gene07426	chr5 (−)	4694929	4697150	8	373	43.5	7.8	33.4
*FAD3* (10700)	Hiprha1gene10700	chr11 (+)	13049184	13051234	8	381	44.1	8.8	30.3
*FAD3* (24146) *	Hiprha1gene24146	chr11 (+)	13199761	13207375	8	315	36.0	6.9	38.4
*FAD3* (24879)	Hiprha1gene24879	chr2 (−)	6129781	6132620	8	372	43.5	8.7	34.0
*FAD6* (25786)	Hiprha1gene25786	chr3 (+)	13421039	13426311	10	442	51.3	9.5	45.8
*FAD7/8* (02511)	Hiprha1gene02511	chr12 (−)	4505441	4508195	8	447	51.0	9.3	39.3
*FAD7/8* (07747)	Hiprha1gene07747	chr7 (−)	52496846	52499330	7	427	48.6	9.1	37.6
*FAD7/8* (17598)	Hiprha1gene17598	chr3 (+)	210546	212500	8	427	48.9	9.0	40.0

Note: * The errors in the annotation of these genes in the *H. rhamnoides* genome ID CNP0001846 are likely present. Chr.—chromosome; Ex.—number of exons; MW—protein molecular weight; pI—protein theoretic isoelectric point; II—protein instability index; “+”—forward strand; “−”—reverse strand.

## Data Availability

The raw sequencing data have been deposited in the NCBI Sequence Read Archive (SRA) under the BioProject accession number PRJNA1163394.

## Supplementary Materials

The following supporting information can be downloaded at: https://www.mdpi.com/article/10.3390/plants13243486/s1. Supplementary File S1: Table S1: CDS sequences of *KAS II*, *FAT*, *SAD*, and *FAD* genes in *Hippophae rhamnoides*; Table S2: Sequences of predicted motifs of KAS II, FAT, SAD, and FAD proteins in *Hippophae rhamnoides*; Table S3: Segmental replication and tandem repeat replication covariate gene pair Ka/Ks analysis of *KAS II*, *FAT*, *SAD*, and *FAD* genes in *Hippophae rhamnoides*; Table S4: CPM values for *KAS II*, *FAT*, *SAD*, and *FAD* genes in seeds and pulp at four fruit ripening stages and leaves of *Hippophae rhamnoides*; Table S5: *Arabidopsis thaliana* genes of the *KAS II*, *FAT*, *SAD*, and *FAD* families used for protein BLAST analysis against the annotated *Hippophae rhamnoides* genome; Supplementary File S2: Figure S1: Distribution of *KAS II*, *FAT*, *SAD*, and *FAD* genes on twelve chromosomes of *Hippophae rhamnoides*; Figure S2: Exon–intron structures of *KAS II*, *FAT*, *SAD*, and *FAD* genes in *Hippophae rhamnoides* and *Arabidopsis thaliana*; Figure S3: Structures of conserved motifs of *KAS II*, *FAT*, *SAD*, and *FAD* genes in *Hippophae rhamnoides*; Figure S4: Conserved Pfam domains of *KAS II*, *FAT*, *SAD*, and *FAD* genes in *Hippophae rhamnoides* and *Arabidopsis thaliana*.
