# Supplementary material for: Identification and Analysis of KAS II, FAT, SAD, and FAD Gene Families in Hippophae rhamnoides
Source: Plants (Basel). 2024 Dec 13;13(24):3486. doi: 10.3390/plants13243486 (PMC11728709; doi:10.3390/plants13243486)
Supplement: Supplementary file 1 [file plants-13-03486-s001.zip › Supplementary_File_S2_2024.12.05.pptx]

## Slide 1
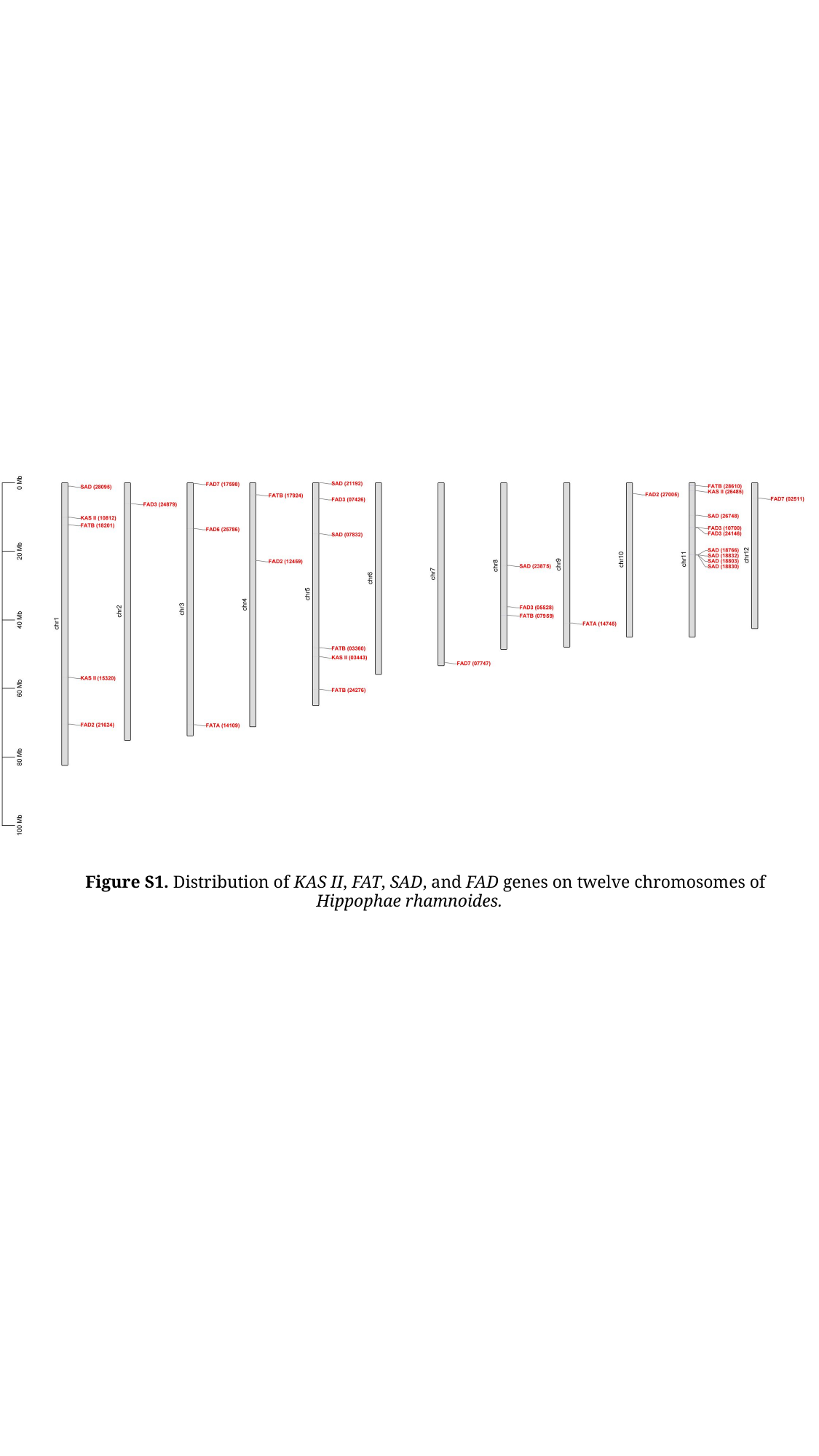

Figure S1. Distribution of KAS II, FAT, SAD, and FAD genes on twelve chromosomes of Hippophae rhamnoides.

## Slide 2
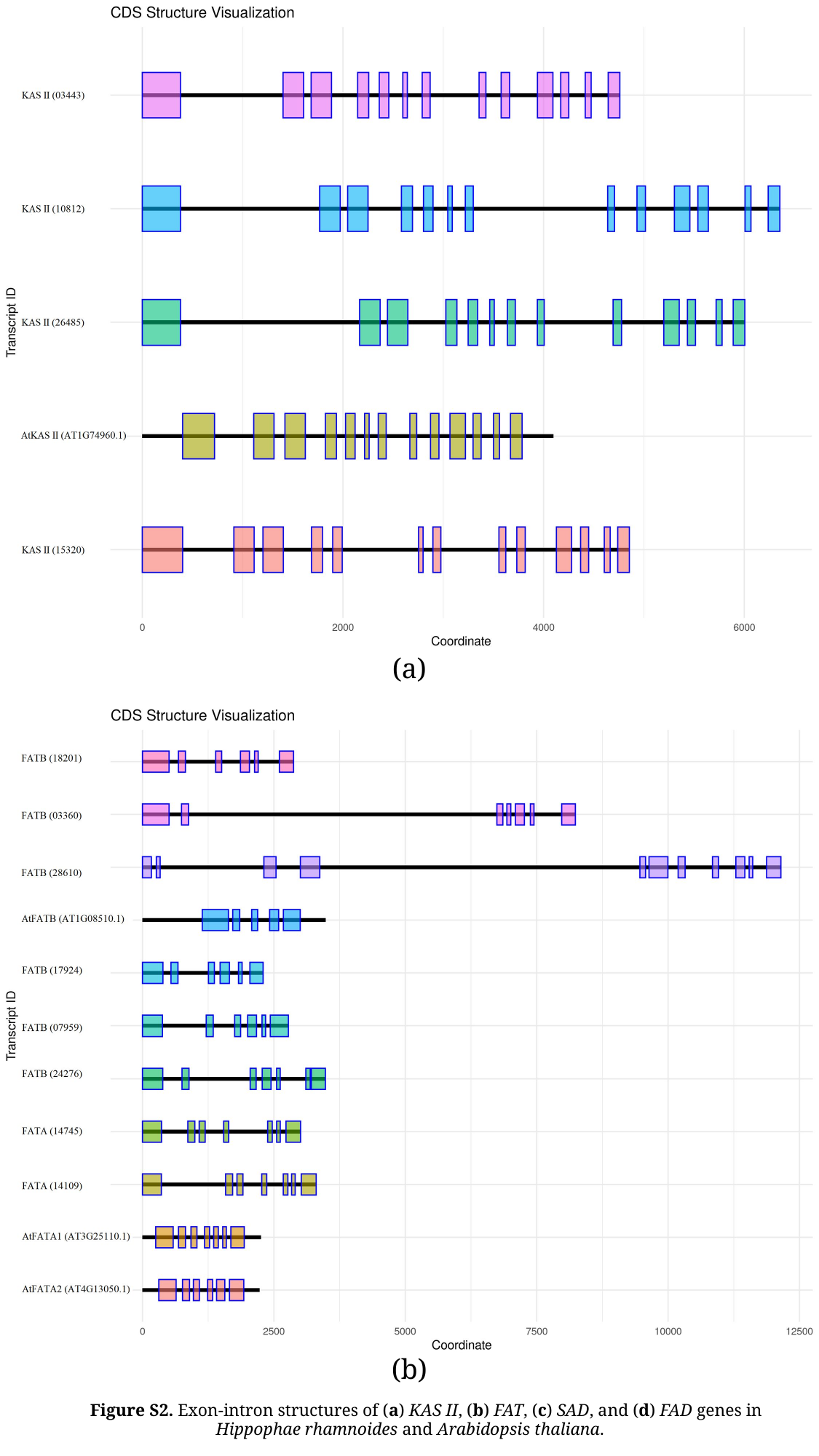

(a)
(b)
Figure S2. Exon-intron structures of (a) KAS II, (b) FAT, (c) SAD, and (d) FAD genes in Hippophae rhamnoides and Arabidopsis thaliana.

## Slide 3
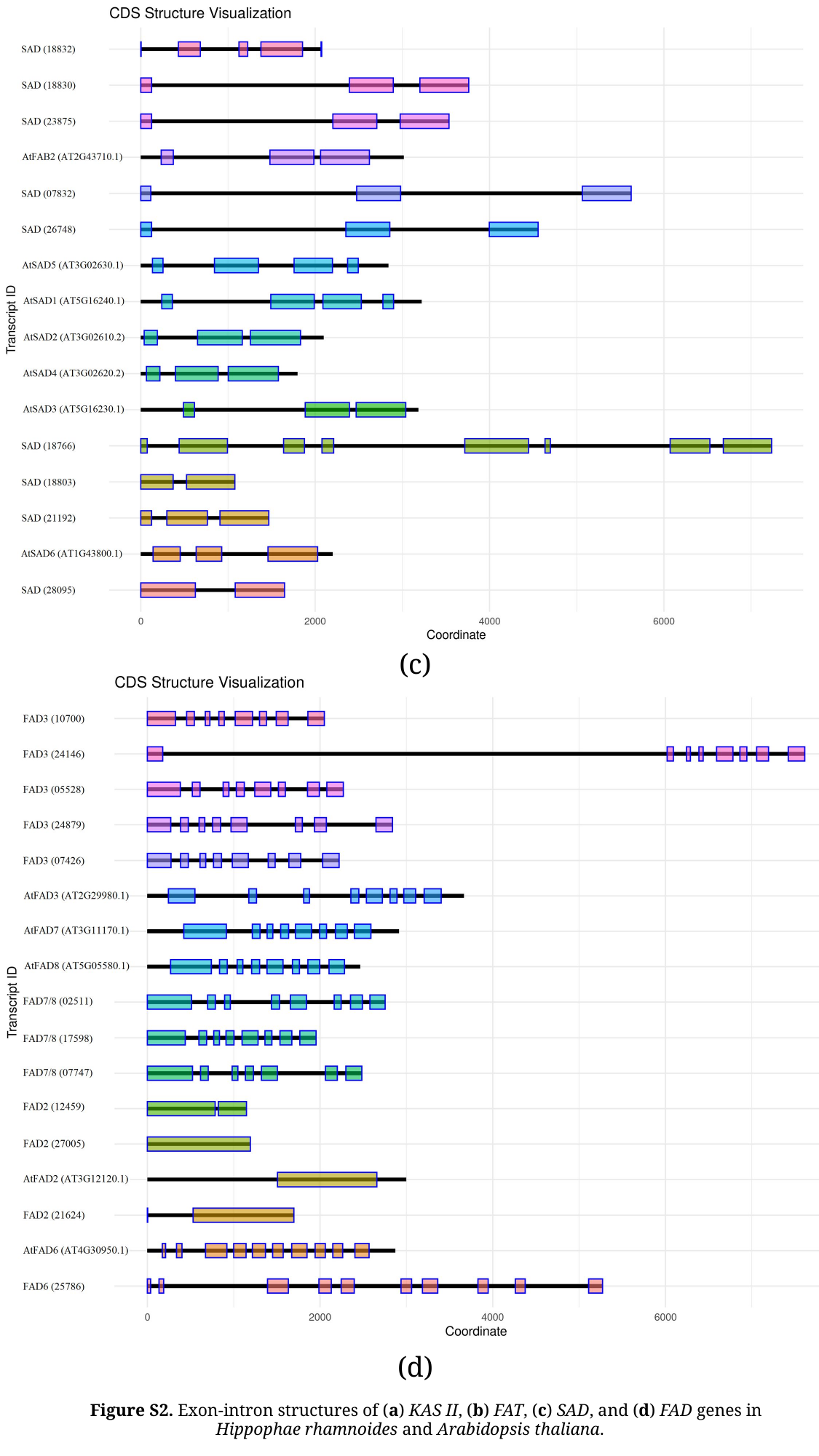

(c)
(d)
Figure S2. Exon-intron structures of (a) KAS II, (b) FAT, (c) SAD, and (d) FAD genes in Hippophae rhamnoides and Arabidopsis thaliana.

## Slide 4
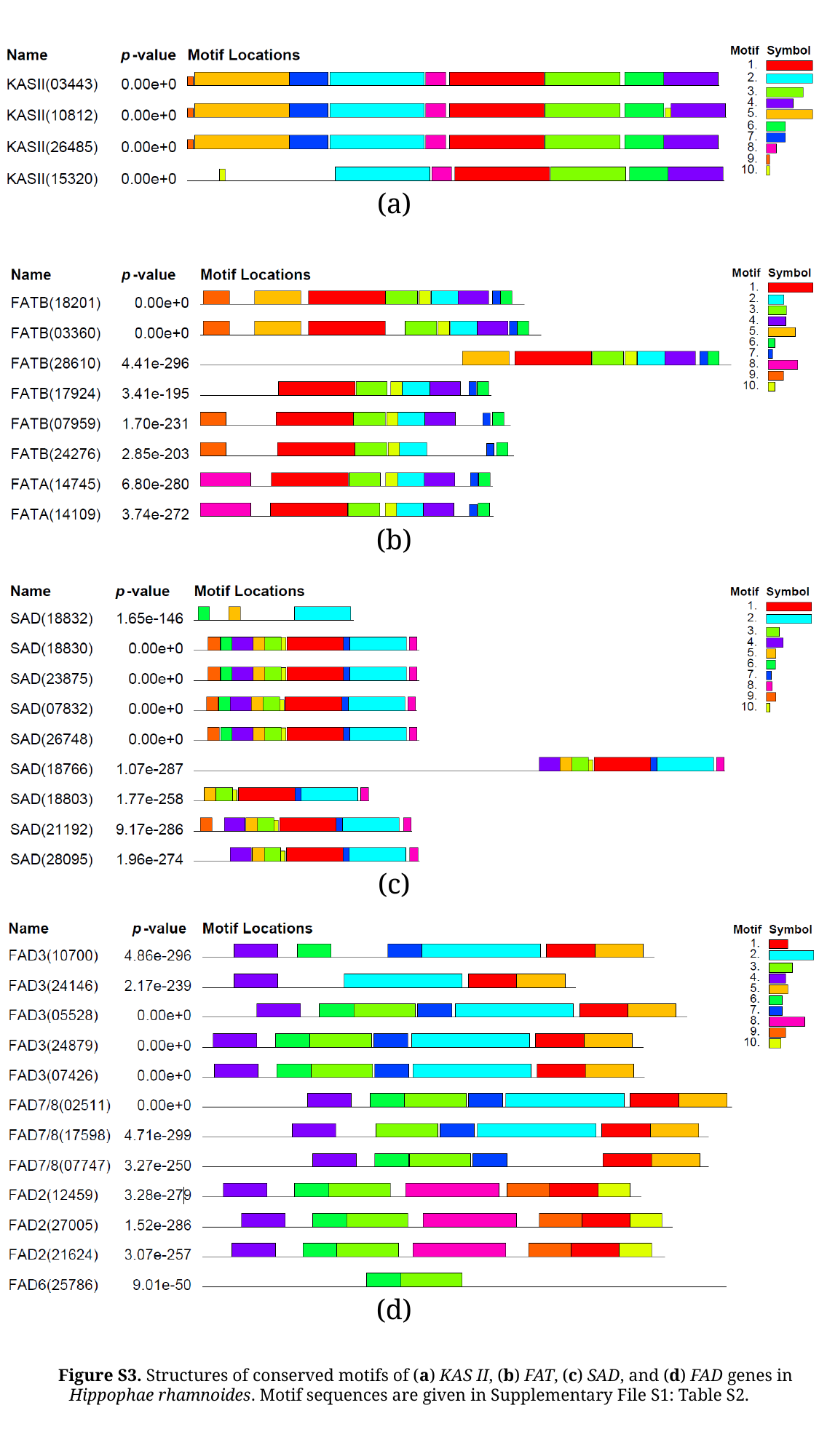

(a)
(b)
(c)
(d)
Figure S3. Structures of conserved motifs of (a) KAS II, (b) FAT, (c) SAD, and (d) FAD genes in Hippophae rhamnoides. Motif sequences are given in Supplementary File S1: Table S2.

## Slide 5
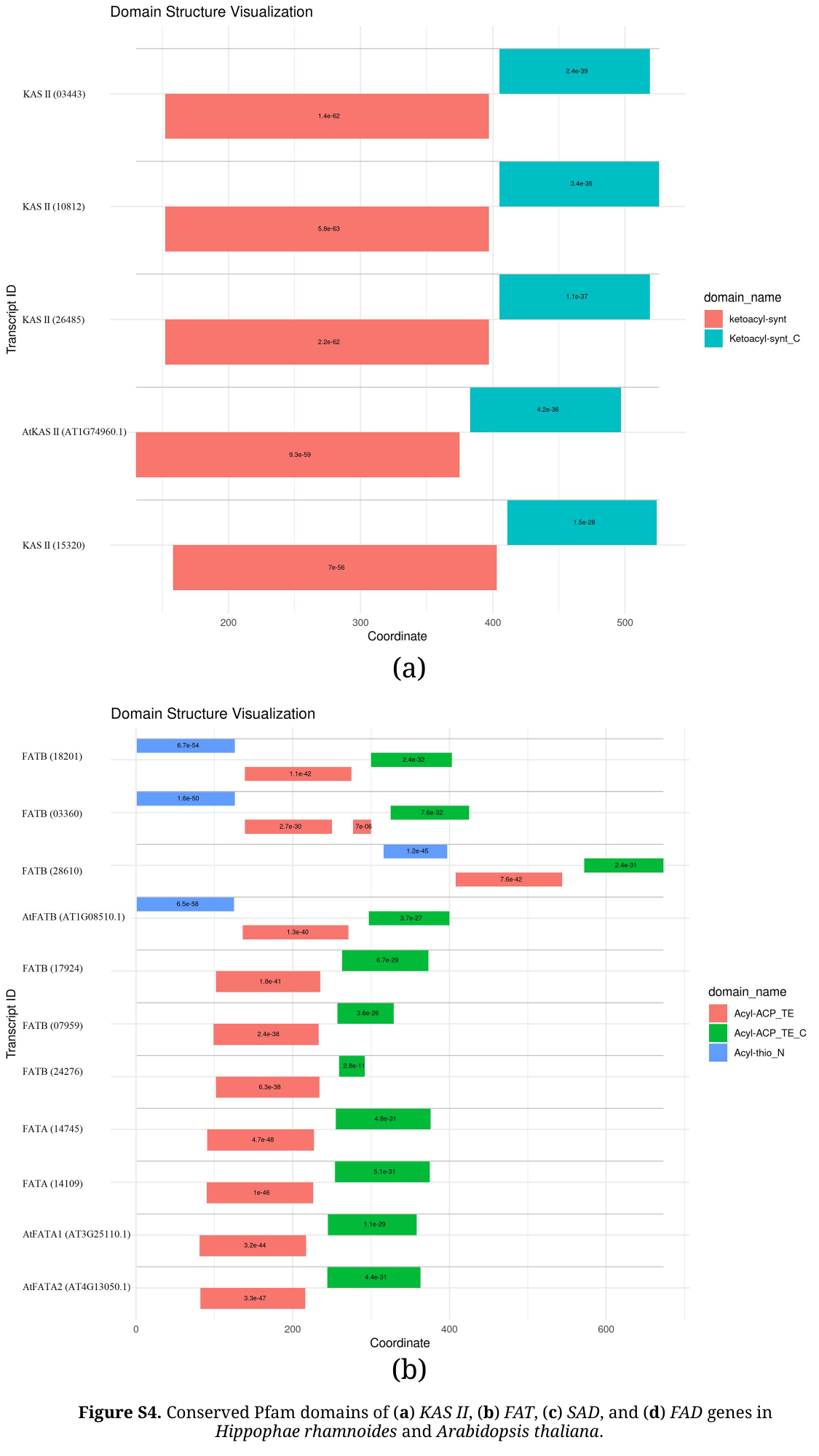

(a)
(b)
Figure S4. Conserved Pfam domains of (a) KAS II, (b) FAT, (c) SAD, and (d) FAD genes in Hippophae rhamnoides and Arabidopsis thaliana.

## Slide 6
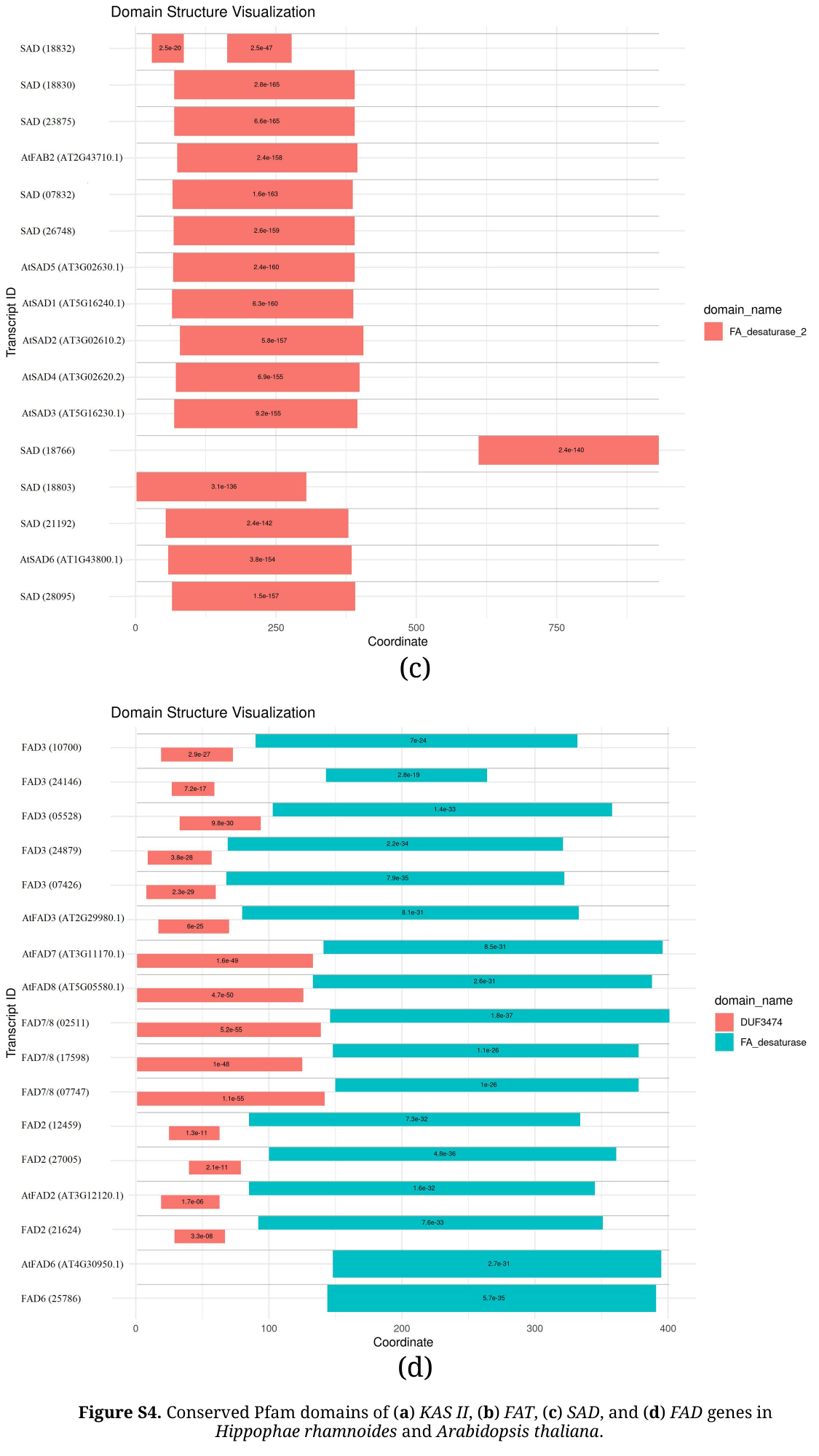

(c)
(d)
Figure S4. Conserved Pfam domains of (a) KAS II, (b) FAT, (c) SAD, and (d) FAD genes in Hippophae rhamnoides and Arabidopsis thaliana.
